# Evaluation of Antibiotic Use for Dental-Related Infections in Dental Clinics Associated With an Academic Safety Net Institution

**DOI:** 10.1093/ofid/ofae556

**Published:** 2024-09-19

**Authors:** Michael A Deaney, Margaret M Cooper, Timothy C Jenkins, Kimberly A Meyers, Katherine C Shihadeh

**Affiliations:** Department of Pharmacy, Denver Health & Hospital Authority, Denver, Colorado, USA; Department of Pharmacy, Denver Health & Hospital Authority, Denver, Colorado, USA; Department of Medicine—Infectious Disease, Denver Health & Hospital Authority, Denver, Colorado, USA; Department of Dentistry, Denver Health & Hospital Authority, Denver, Colorado, USA; Department of Pharmacy, Denver Health & Hospital Authority, Denver, Colorado, USA

**Keywords:** antibiotic, dental, outpatient, stewardship, surveillance

## Abstract

This retrospective study found lower antibiotic prescribing rates by outpatient dentists than previous literature, but with deviations from guideline recommendations in antibiotic indications and durations of treatment for oral pain and swelling. These findings will guide future stewardship interventions to promote guideline-directed therapy plans.

Dentists working in outpatient clinics frequently prescribe antibiotics for various conditions. Studies indicate that a significant proportion of outpatient antibiotics are prescribed by dentists, with a 2015 analysis ranking dentists as the third highest prescriber by volume [1–3]. Within the Veterans Affairs system, 3.5% of dental clinic visits in 1 calendar year resulted in an antibiotic prescription [4]. Recent evidence highlights a lack of uniformity in dental prescribing practices. A 2018 systematic review revealed variability in drug selection and treatment duration, with many prescriptions issued for non-guideline-directed indications where localized treatments would have sufficed [5].

In 2019, the American Dental Association (ADA) published guidelines on antibiotic use for adults with oral pain and swelling [6, 7]. These guidelines delineate scenarios for antibiotic indications and recommend therapy plans. A 2017 study by Carlson et al. evaluated antibiotic prescribing practices in the VA dental clinics and compared these practices with the 2019 ADA guidelines. The authors found that only 59% of prescriptions utilized the first-line antibiotic, amoxicillin, and durations exceeded the recommendations in 36.5% of cases [4]. Despite dentists’ knowledge of guideline-directed therapies, barriers like time constraints, lack of incentives for localized treatments, patient demand for antibiotics, and concerns about undertreatment impede implementation [8]. This underscores the need for extending antimicrobial stewardship programs to dental clinics to standardize antibiotic prescribing, enhance prescribing awareness, and mitigate side effects, global antibiotic resistance, and costs [9].

The primary objective of this study was to assess antibiotic prescribing practices by dentists in outpatient clinics within the Denver Health system, focusing on overall prescribing rates and concordance with ADA guidelines in terms of indication, antibiotic selection, dose, and duration.

## METHODS

This retrospective cohort study was conducted across 7 dental clinics. Antibiotic prescriptions written by 1 of the 49 actively practicing dentists at Denver Health between July 1, 2022, and November 1, 2023, were identified from our health care data warehouse. Prescriptions for hospitalized patients, or those by nondental providers, were excluded.

Given the emphasis on prescription volume in the existing literature, the primary outcome was the rate of antibiotic prescriptions per dental visit. A random subset of 200 prescriptions was selected for detailed manual chart review using a function in Microsoft Excel, with the sample size determined by manpower limitations. Key secondary outcomes for this subset included (1) the proportion of antibiotic prescriptions concordant with ADA guideline recommendations for antibiotic indication, selection, dose, and duration and (2) the proportion of patients with a low-risk penicillin allergy who were treated with a second-line antibiotic, with a low-risk allergy defined as a PEN-FAST score of <3, indicating a <1%–5% chance of a positive penicillin allergy test [10]. Prescriptions intended for prophylaxis before a procedure or for a pediatric patient were excluded from this analysis as they utilized different guidelines than the 2019 ADA recommendations.

For treatment of oral pain and swelling, indications were considered guideline-directed if they aligned with ADA recommendations, specifically requiring documentation of 3 clinical criteria: oral pain and swelling, apical abscess, and pulp necrosis. If direct, conservative dental treatment (DCDT) was performed within 2 days of prescription, documentation of fever or malaise was also required to be guideline-directed. Other appropriate indications included postoperative swelling and pericoronitis. Cases lacking documentation of these characteristics were classified as “indeterminate” in meeting criteria.

ADA guideline-directed antibiotic regimens included amoxicillin 500 mg 3 times daily for 3–7 days as first-line therapy. In cases of a severe penicillin allergy, azithromycin 500 mg on day 1 followed by 250 mg for 4 days, or clindamycin 300 mg 4 times daily for 3–7 days, was considered guideline-directed. Amoxicillin or cephalexin 500 mg 4 times daily for 3–7 days could be used in cases of lower risk penicillin allergies. If patients failed an adequate course of first-line therapy, adding metronidazole 500 mg 3 times daily or switching to amoxicillin-clavulanate 500–125 mg 3 times daily for 7 days was guideline-directed. For follow-up, the ADA recommends that patients be re-evaluated within 3 days and instructed to discontinue antibiotics 24 hours after symptoms resolve. Progress notes and prescription directions were reviewed for adherence to these recommendations.

## RESULTS

A total of 1874 antibiotic prescriptions were written during 114 231 clinic visits, for an antibiotic prescribing rate of 1.6%.

Of the 200 randomly selected patients, 4 (2%) were excluded for receiving prophylactic antibiotics (2%) and a further 12 (6.1%) for being under 18 years of age. The remaining 184 (92%) were adult outpatients prescribed antibiotics for the treatment of oral pain and swelling. The details of these cases are outlined in Table 1. The median patient age was 47, with few patients having comorbid conditions that increase risk of infections like diabetes mellitus (13%) or an immunocompromising condition (2.2%).

**Table 1.** Baseline Demographics, Dental Diagnosis, and Antibiotic Regimens for Those Receiving Antibiotics for Treatment of a Dental Condition (n = 184)

Age	Median (IQR), y	47 (33–56)
Sex assigned at birth	Female, No. (%)	116 (63)
	White or Caucasian, No. (%)	91 (49.5)
	Black or African American, No. (%)	44 (23.9)
	Other, No. (%)	42 (22.8)
	Declined to answer	4 (2.2)
	Asian or Pacific Islander	3 (1.6)
Ethnicity	Hispanic	91 (49.5)
	Non-Hispanic	93 (50.5)
Comorbidities	Diabetes mellitus, No. (%)	24 (13)
	Immunocompromised,^a^ No. (%)	4 (2.2)
	Chronic kidney disease, No. (%)	6 (3.3)
	Cirrhosis, No. (%)	2 (1.1)
Clinical diagnosis	Irreversible pulpitis with or without periodontitis assessment, No. (%)	29 (15.8)
	Pulp necrosis with apical abscess, No. (%)	29 (15.8)
	Postoperative swelling, No. (%)	24 (13)
	Caries, No. (%)	23 (12.5)
	Apical abscess without pulp assessment, No. (%)	20 (10.8)
	Periodontitis without pulp assessment, No. (%)	12 (6.5)
	Pulp necrosis with apical periodontitis, No. (%)	10 (5.4)
	Fractured teeth, No. (%)	6 (3.3)
	Nonrestorable tooth, No. (%)	4 (2.2)
	Periodontal scaling and root tip, No. (%)	4 (2.2)
	Pericoronitis, No. (%)	4 (2.2)
	Dental pain, No. (%)	3 (1.6)
	Dental trauma, No. (%)	2 (1.1)
	Calculus, No. (%)	2 (1.1)
	Other, No. (%)	12 (6.5)
Plan for DCDT	<2 d, No. (%)	45 (24.5)
	>2 d, No. (%)	115 (62.5)
	Not planned or performed	24 (13)
Pain	Present, No. (%)	157 (85.4)
	Absent, No. (%)	24 (13)
	Not documented, No. (%)	3 (1.6)
	Documented pain scale score, median (IQR)	6 (4–9)
Swelling	Present, No. (%)	51 (27.7)
	Absent, No. (%)	74 (40.2)
	Not documented, No. (%)	59 (32.1)
Systemic signs of infection (ie, fever, malaise)	Not documented, No. (%)	184 (100)
Antibiotic selection	Amoxicillin, No. (%)	144 (78.3)
	Clindamycin, No. (%)	18 (9.8)
	Amoxicillin-clavulanate, No. (%)	15 (8.2)
	Penicillin, No. (%)	5 (2.7)
	Azithromycin, No. (%)	1 (0.5)
	Doxycycline, No. (%)	1 (0.5)
Antibiotic duration	3 d, No. (%)	2 (1.1)
	5 d, No. (%)	26 (14.1)
	7 d, No. (%)	114 (62)
	10 d, No. (%)	41 (22.3)
	>10 d, No. (%)	1 (0.5)
	Median (IQR)	7 (7–7)
Instructions to discontinue antibiotics 24 h after symptom resolution	Not documented, No. (%)	184 (100)

Abbreviations: DCDT, direct, conservative dental treatment; IQR, interquartile range;

^a^Immunocompromised was defined as concomitant immunosuppressive conditions like hematologic malignancies and HIV with a CD4 count <200 or the use of corticosteroids, chemotherapeutics, transplant rejection medications, or other medications that suppress the immune system.

One of the most common clinical diagnoses was pulp necrosis with apical abscess (15.8%), a guideline-directed indication. Others included irreversible pulpitis, either alone or with associated periodontitis (15.8%), and a general diagnosis of “caries” (12.5%). Most (62.5%) treatment plans included receiving DCDT >2 days after prescription. Pain was documented in most (85.4%) cases, with a median pain score of 6. Swelling was either absent (40.2%) or undocumented (32.1%) in the medical record of most cases. Neither fever nor malaise was documented in any case.

The prescribed antibiotics were predominantly amoxicillin (78.3%), followed by clindamycin (9.8%) and amoxicillin-clavulanate (8.2%). The most common treatment duration was 7 days (62%), followed by 10 days (22.3%). No cases had documented instructions to discontinue antibiotics within 24 hours of symptom resolution.

Evaluation of individual cases showed that very few met all the criteria for guideline-directed therapy (3.3%) (Table 2). A significant proportion had at least 1 criterion that made them discordant with guideline recommendations (66.8%). The remaining 29.9% of cases had guideline-directed antibiotic regimens but were missing documentation regarding key clinical variables for evaluation of indication (ie, pain, swelling, systemic signs of infection) and were deemed indeterminate.

**Table 2.** Proportion of Guideline-Directed Antimicrobial Regimens for Treatment of Dental Conditions (n = 184)

Characteristic	Guideline-Directed Prescriptions, No. (%)
Antibiotic indication, selection, dose, and duration	6 (3.3)
Antibiotic indication	9 (4.9)
Antibiotic selection	150 (81.5)
Antibiotic dose	177 (96.2)
Antibiotic duration	142 (77.2)

Of the 18 prescriptions for clindamycin, 16 were administered to patients with documented penicillin allergies. Of these, 87.5% (n = 14) had a PEN-FAST score of <3. One case of *Clostridioides difficile* infection was captured by the electronic medical record within 30 days of antibiotic prescription, occurring in a patient who received clindamycin.

## CONCLUSIONS

This retrospective cohort study evaluated antibiotic prescribing by dentists, revealing a prescribing rate of 1.6%, less than the 3.5% reported by Carlsen et al. [2]. This difference may reflect variations in patient population, clinician factors, organizational culture, or increased awareness of antibiotic guidelines in dentistry [6, 11].

In a random subset of 184 patients, inconsistencies were observed in the indications for antibiotic use, with only 9 cases (4.9%) documenting all characteristics outlined by the guidelines. Prescriptions for apical abscesses with pulp necrosis were guideline-directed, but an equal number were issued for irreversible pulpitis, which is not guideline-directed. Cases diagnosed as “caries” might have met criteria with more thorough documentation of pulp status or abscess involvement. Overall, 47.8% of cases did not meet criteria, often due to documented absence of pain or swelling. The remaining cases (47.3%) were classified as “indeterminate” due to lack of documentation of key guideline criteria, like swelling or systemic signs of infection.

Guideline-directed antibiotic selection and duration were higher than those observed by Carlsen et al., though around 20% of cases were still discordant with guideline recommendations. Many non-guideline-directed selections were for patients with penicillin allergies, with clindamycin chosen more often than azithromycin. The incidence of *C. difficile* infection following a clindamycin prescription demonstrates the potential risks of this practice. Most penicillin allergies were low risk, indicating that amoxicillin or cephalexin could have been appropriate choices. Regarding duration, 77.2% of cases fell within the guideline-directed 3–7 days, with 62% prescribed for 7 days. Although this is guideline-directed, there is opportunity to shorten durations to 3–5 days to promote the shortest effective duration. Enhanced documentation of follow-up and instructions to discontinue antibiotics after symptom resolution could further improve guideline adherence. Antibiotic dose often aligned with guideline recommendations.

This is the first study comparing dental prescribing practices with the 2019 ADA guidelines after their publication. However, its generalizability is limited by its single health care system setting, which includes a dental residency program that may increase exposure to recent guidelines.

Despite these limitations, this study identifies key areas for antimicrobial stewardship, including (1) improving documentation of criteria for which an antibiotic would be indicated, (2) reducing duration of therapy by emphasizing 3–5-day durations and instructing patients to discontinue antibiotics after symptom resolution, and (3) selecting appropriate antibiotics in low-risk penicillin allergies. Future efforts to improve antibiotic prescribing from dental clinics will involve a collaborative, multifaceted intervention to be implemented over the next year.

In conclusion, while antibiotic prescribing rates among dental providers are consistent with or lower than existing literature, significant opportunities exist to enhance guideline adherence by using guideline-directed treatment regimens and improving documentation practices. Continued evaluation and targeted stewardship interventions are needed to address these gaps.

## References

[1] Hicks LA, Bartoces MG, Roberts RM, et al US outpatient antibiotic prescribing variation according to geography, patient population, and provider specialty in 2011. Clin Infect Dis 2015; 60:1308–16. 25747410 10.1093/cid/civ076

[2] Suda KJ, Roberts RM, Hunkler RJ, Taylor TH. Antibiotic prescriptions in the community by type of provider in the United States, 2005–2010. J Am Pharm Assoc 2016; 56:621–6.e1. 10.1016/j.japh.2016.08.015 27745794

[3] Durkin MJ, Hsueh K, Sallah YH, et al An evaluation of dental antibiotic prescribing practices in the United States. J Am Dent Assoc 2017; 148:878–86.e1. 28941554 10.1016/j.adaj.2017.07.019 PMC5705569

[4] Carlsen DB, Durkin MJ, Gibson G, et al Concordance of antibiotic prescribing with the American Dental Association acute oral infection guidelines within Veterans’ Affairs (VA) dentistry. Infect Control Hosp Epidemiol 2021; 42:1422–30. 33650474 10.1017/ice.2021.16 PMC8410877

[5] Stein K, Farmer J, Singhal S, Marra F, Sutherland S, Quiñonez C. The use and misuse of antibiotics in dentistry: a scoping review. J Am Dent Assoc 2018; 149:869–84.e5. 30261952 10.1016/j.adaj.2018.05.034

[6] Lockhart PB, Tampi MP, Abt E, et al Evidence-based clinical practice guideline on antibiotic use for the urgent management of pulpal- and periapical-related dental pain and intraoral swelling: a report from the American Dental Association. J Am Dent Assoc 2019; 150:906–21.e12. 31668170 10.1016/j.adaj.2019.08.020 PMC8270006

[7] American Academy of Pediatric Dentistry . Use of antibiotic therapy for pediatric dental patients. In: The Reference Manual of Pediatric Dentistry. Chicago, IL: American Academy of Pediatric Dentistry; 2023:537–41.

[8] Newlands R, Duncan EM, Prior M, et al Barriers and facilitators of evidence-based management of patients with bacterial infections among general dental practitioners: a theory-informed interview study. Implement Sci 2016; 11:11. 26821790 10.1186/s13012-016-0372-z PMC4731984

[9] American Dental Association . Antibiotic stewardship. www.ada.org. Published April 5, **2023**. Available at: https://www.ada.org/resources/research/science-and-research-institute/oral-health-topics/antibiotic-stewardship. Accessed August 4, 2023.

[10] Copaescu AM, Vogrin S, James F, et al Efficacy of a clinical decision rule to enable direct oral challenge in patients with low-risk penicillin allergy: the PALACE randomized clinical trial. JAMA Intern Med 2023; 183:944–52. 37459086 10.1001/jamainternmed.2023.2986 PMC10352926

[11] Wilson WR, Gewitz M, Lockhart PB, et al Prevention of viridans group streptococcal infective endocarditis: a scientific statement from the American Heart Association. Circulation 2021; 143:e963–78. 33853363 10.1161/CIR.0000000000000969

